# Impact of Coating of Urea with *Bacillus*-Augmented Zinc Oxide on Wheat Grown under Salinity Stress

**DOI:** 10.3390/plants9101375

**Published:** 2020-10-15

**Authors:** Noor Ul Ain, Muhammad Naveed, Azhar Hussain, Muhammad Zahid Mumtaz, Munazza Rafique, Muhammad Asaad Bashir, Saud Alamri, Manzer H. Siddiqui

**Affiliations:** 1 Institute of Soil and Environmental Sciences, University of Agriculture, Faisalabad 38040, Pakistan; aini.noorulain@gmail.com; 2 China Pakistan Economic Corridor Unit/World Trade Organization Cell, Agriculture Department, Government of Punjab, Lahore 54000, Pakistan; 3 Department of Soil Science, The Islamia University of Bahawalpur, Bahawalpur 63100, Pakistan; m.asaadbashir@gmail.com; 4 Institute of Molecular Biology and Biotechnology, The University of Lahore, Main Campus, Defense Road, Lahore 54000, Pakistan; zahidses@gmail.com; 5 Soil Bacteriology Section, Agricultural Biotechnology Research Institute, Ayub Agriculture Research Institute, Faisalabad 38000, Pakistan; munazzaaari@gmail.com; 6 Department of Botany and Microbiology, College of Science, King Saud University, Riyadh 11451, Saudi Arabia; saualamri@ksu.edu.sa (S.A.); manzerhs@yahoo.co.in (M.H.S.)

**Keywords:** *Bacillus* spp., physiology, salinity, *Triticum aestivum*, zinc-coated urea, zinc uptake

## Abstract

Zinc (Zn) availability is limited in salt-affected soils due to high soil pH and calcium concentrations causing Zn fixation. The application of synthetic Zn fertilizer is usually discouraged due to the high cost and low Zn use efficiency. However, salt-tolerant Zn-solubilizing bacteria (ZSB) are capable of solubilizing fixed fractions of Zn and improving fertilizer use efficiency. In the current study, a product was formulated by coating urea with bioaugmented zinc oxide (ZnO) to improve wheat productivity under a saline environment. The promising ZSB strain *Bacillus* sp. AZ6 was used for bioaugmentation on ZnO powder and termed as *Bacillus* sp. AZ6-augmented ZnO (BAZ). The experiment was conducted in pots by applying urea granules after coating with BAZ, to evaluate its effects on wheat physiology, antioxidant activity, and productivity under saline (100 mM NaCl) and non-saline (0 mM NaCl) conditions. The results revealed that the application of BAZ-coated urea alleviated salt stress through improving the seed germination, plant height, root length, photosynthetic rate, transpiration rate, stomatal conductance, soil plant analysis development (SPAD) value, number of tillers and grains, spike length, spike weight, 1000-grain weight, antioxidant activity (APX, GPX, GST, GR, CAT, and SOD), and NPK contents in the straw and grains of the wheat plants. Moreover, it also enhanced the Zn contents in the shoots and grains of wheat by up to 29.1 and 16.5%, respectively, over absolute control, under saline conditions. The relationships and variation among all the studied morpho-physio and biochemical attributes of wheat were also studied by principal component (PC) and correlation analysis. Hence, the application of such potential products may enhance nutrient availability and Zn uptake in wheat under salt stress. Therefore, the current study suggests the application of BAZ-coated urea for enhancing wheat’s physiology, antioxidant system, nutrient efficiency, and productivity effectively and economically.

## 1. Introduction

Global food production is set to keep growing, even with a projected decline in total arable land over time [1]. The reduction in arable lands is the result of various factors including climate change, urban encroachment, and abiotic stresses. Among abiotic stresses, an increase in soil salinity is a serious and global threat to agricultural production. The rise in salt-affected soils could be due to an excess of soluble salts known as saline soils, while the supremacy of exchangeable sodium (Na^+^) in soil is termed as sodic soils, or a mixture of both situations is called saline-sodic soils [2]. According to an estimate, the increase in the total area of salt-affected land from 1986 to 2016 was around 1 billion hectares [3]; that might be due to low rainfall, high evapotranspiration, defective drainage, and/or the successive application of fertilizers, soil amendments, and irrigation water having high salt contents [4].

Salinity damages plant growth through osmotic and ionic stresses [5]. Osmotic stress is caused by the inhibition of water absorption, cell elongation, stomatal conductance, and the accumulation of salts in terms of Na^+^ and chloride (Cl^−^) that result in ionic-stress [6]. Ionic stress causes a reduction in potassium (K^+^) uptake and leads to oxidative stress through leaf senescence. It also damages the proteins, lipids, DNA, and cellular functions, and inhibits enzymatic and photosynthetic activity through reactive oxygen species (ROS) production [7,8]. Plants usually adopt three main mechanisms of action to tolerate salinity stress: (a) ion exclusion, in which the Na^+^ transporter lessens the gathering of lethal Na^+^ inside roots, (b) promoting tissue tolerance through the compartmentalization of toxic Na^+^ ions into specific tissues, and (c) sustaining growth and water uptake despite Na^+^ gathering in shoots [5]. Various physiological components including photosynthesis, transpiration use efficiency, and the production of antioxidants contribute to salinity tolerance [7,9].

Zinc (Zn) is an essential micronutrient for plants and occurs as a free ion or complexed with low molecular weight organic compounds [10]. It acts as a catalyst and cofactor in hundreds of enzymes and proteins, including Zn finger protein [11]. Cereals are intrinsically low in Zn contents, and unfortunately, populations consuming cereals are facing widespread micronutrient malnutrition, over the globe [12,13,14]. Zn deficiency is observed in plants grown under calcareous and salt-affected soils that not only damage chloroplast structure but also reduce photosynthesis (by up to 50–70%) and cause toxicity from ROS [15]. According to Amiri et al. [16], the application of Zn can alleviate salt stress through promoting physiological attributes including photosynthesis, stomatal conductance, proline contents, photosystem II photochemistry, and superoxide dismutase (SOD) activity in almond seedlings. Another new approach to alleviating salinity stress recommended by Azarmi et al. [17] is the application of Zn and plant growth-promoting rhizobacteria (PGPR). They reported that the combined application of Zn and 1-aminocyclopropane-1-carboxylate (ACC)-deaminase-containing PGPR significantly promoted antioxidant enzyme activity and protein concentration at 2000 mg kg^−1^ of NaCl-salt stress.

The application of PGPR as a bioinoculant has the potential to improve plant growth and development under salt-stress conditions by producing plant growth-promoting (PGP) substances in the form of their secondary metabolites [18]. Among such PGPR, *Bacillus* spp. is one of the most effective PGP agents due to its ability to produce various PGP metabolites and increase plant growth under salt stress [19]. Such PGPR can counteract the harmful effects of salt stress via lowering ethylene production by producing ACC-deaminase enzymes and induce salt tolerance in plants [18]. Due to their adaptive mechanism, such bioinoculants are being applied to improve crop productivity and soil health. Bioinoculants with macro- and micronutrient applications showed sustainable results and increased fertilizer efficiency in salt-affected soils [20]. Soil-applied inorganic micronutrients, especially Zn, become unavailable soon after their application. Zn is mostly found in soil in various insoluble forms including zinc oxide (ZnO); however, its availability depends on the weathering of parent minerals and atmospheric contribution of Zn dissolution in soils [21]. However, nano-structured ZnO showed greater dissolution and promoted crop productivity and Zn uptake in plants [22]. Zn-solubilizing rhizobacteria showed their power to dissolve bulk ZnO powder through producing organic acids [23,24]. Recently, Hussain et al. [25,26] reported the application of bioactivated zinc oxide (ZnO) augmented with *Bacillus* sp. AZ6 to boost maize productivity. They reported a sustainable increase in crop productivity by improving plant physiology and metabolism. Such a novel biotechnological approach may be adopted under abiotic stresses. Following the above facts, the current study was conducted to improve wheat productivity through the application of urea coated with bioaugmented ZnO under salinity stress. A urea-coated product was developed by augmenting ZnO with *Bacillus* sp. AZ6 to evaluate its effect on wheat physiology, antioxidant activity, nutrient efficiency, and productivity under salt stress.

## 2. Results

### 2.1. Soil Characterization

A pot study was performed to evaluate the effects of BAZ-coated urea on wheat performance under salinity stress. The physicochemical characteristics of the pot soil used to produce an artificial salinity of 100 mM NaCl before conducting the experiment were analyzed following standard procedures. The experimental soil was sandy clay loam having 49.8% sand, 30.2% silt, and 20% clay contents. The soil showed a saturation percentage of 28%, pH of 7.96, and electrical conductivity (EC) of 1.49 dS m^−1^. The fertility of the soil revealed that it contained a low amount of organic matter (<1%), 0.051% total N, 8.79 mg kg^−1^ available P, 84 mg kg^−1^ extractable K, and 0.51 mg kg^−1^ available Zn.

### 2.2. Physiological Attributes of Wheat

**Figure 1:**
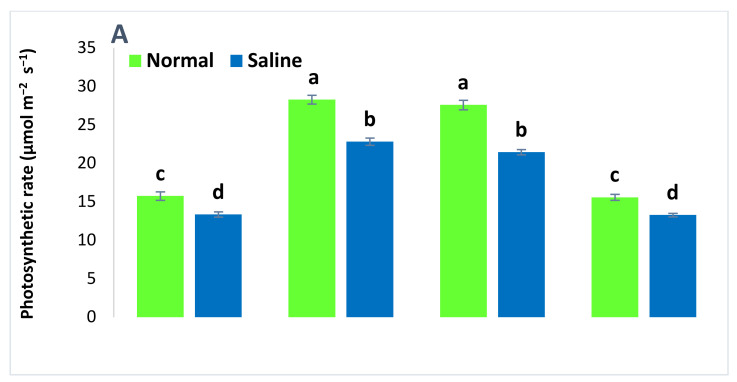

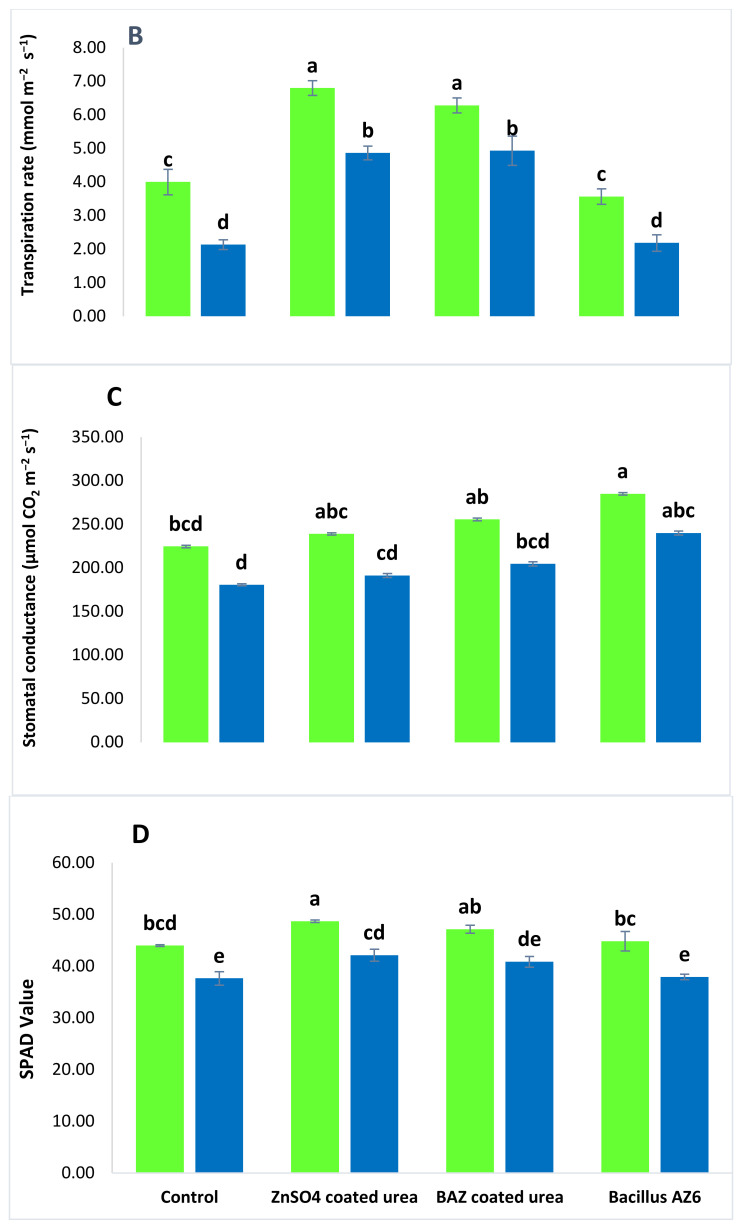
The photosynthetic rate (**A**), transpiration rate (**B**), stomatal conductance (**C**), and soil plant analysis development (SPAD) value (**D**) of wheat grown with BAZ (*Bacillus* augmented ZnO)-coated urea under salinity stress. These physiological attributes were observed at the flowering stage, and the data presented here are the means of three replications (*n* = 3) ± standard error, having three plants in each replication. Different alphabetical letters above error bars show significant differences (*p* ≤ 0.05) among different treatments.

The addition of salt stress (100 mM) caused a significant reduction in the physiological attributes of wheat (Figure 1). Saline conditions reduced the photosynthetic rate, transpiration rate, stomatal conductance, and soil plant analysis development (SPAD) value in all the treatments as compared to non-saline conditions. The application of both ZnSO_4_ and BAZ-coated urea alleviated the salt stress and significantly promoted wheat’s physiological attributes. These treatments were not significantly different to each other under saline and non-saline conditions; however, these treatments were significantly different from the absolute controls for the respective saline and non-saline conditions. The BAZ-coated urea application increased the photosynthetic rate, transpiration rate, stomatal conductance, and SPAD value by up to 37.8%, 56.7%, 46.6%, and 7.8%, respectively, as compared to absolute controls under saline conditions. Meanwhile, under non-saline conditions, this treatment caused increases of 42.9% and 36.3% in the photosynthetic and transpiration rates, respectively, as compared to absolute control. The increase in stomatal conductance and SPAD value due to BAZ-coated urea was non-significant under the saline condition. The results also revealed the increase in wheat physiology due to inoculation with *Bacillus* sp. AZ6 was non-significant with respect to absolute control under both saline and non-saline conditions (Figure 1).

### 2.3. Improvement in Growth Attributes

The data regarding the influence of BAZ-coated urea on wheat growth in saline and non-saline conditions are shown in Table 1. The results revealed that salt stress caused a significant reduction in wheat growth attributes such as seed germination, plant height, root length, and the number of tillers in all the treatments, as compared to the salt-stress-free conditions. The inoculation of the *Bacillus* sp. strain AZ6 caused a significant increase in wheat germination and plant height under both saline and non-saline conditions; however, the increase in root length and the number of tillers due to strain AZ6 was statistically similar to the respective absolute controls for saline and non-saline conditions. The application of both BAZ-coated urea and ZnSO_4_-coated urea showed a significantly higher increase in wheat growth in saline as well as in non-saline conditions, and the increases were not significantly different from each other but highly significantly different from their respective absolute controls. Under saline conditions, the BAZ-coated urea promoted seed germination by up to 17.9%, plant height by up to 24.6%, root length by up to 17.8%, and the number of tillers by up to 23.3% over absolute control, while, under non-saline conditions, it showed 18.1, 18.3, 17.6, and 17.5% increases in seed germination, plant height, root length, and the number of tillers, respectively, as compared to absolute control.

### 2.4. Effect on Yield Parameters

A significant reduction in yield parameters (the spike length and weight, number of grains, and 1000-grain weight) in all the treatments was observed in plants grown under saline conditions (Table 2). Under saline conditions, BAZ-coated urea significantly promoted the number of grains spike^−1^ by up to 26.3%, spike length by up to 18.6%, spike weight by up to 23.9%, and 1000-grain weight by up to 14.9%, as compared to absolute control. Meanwhile, its application under non-saline conditions promoted the number of grains spike^−1^ by up to 24.5%, spike length by up to 23.4%, spike weight by up to 19.1%, and 1000-grain weight by up to 12.2%, over absolute control. Both in saline and non-saline conditions, an increase in yield attributes due to BAZ-coated urea was statistically similar to that with ZnSO_4_-coated urea; however, both of these treatments were highly significant with respect to absolute control. There was no significant change in the number of grains spike^−1^, spike length and weight, and 1000-grain weight in *Bacillus*-inoculated plants under saline as well as non-saline conditions (Table 3).

### 2.5. Antioxidant Assays

The application of salinity stress caused a significant increase in the antioxidant activities of wheat in all the treatments (Table 3). Saline conditions (100 mM) increased the APX, GPX, GST, GR, CAT, and SOD activity by up to 44, 49, 51, 46, 55, and 41%, respectively, over non-saline conditions. The application of both ZnSO_4_- and BAZ-coated urea alleviated the salt stress and significantly promoted the antioxidant activity of the wheat crop. These treatments were not significantly different from each other under salt-stress and salt-stress-free conditions; however, these treatments were significantly different from the absolute controls for the respective saline and non-saline conditions. BAZ-coated urea application decreased the activity of APX by up to 44%, GPX by up to 42%, GST by up to 41%, GR by up to 37%, CAT by up to 44%, and SOD by up to 39%, as compared to absolute control under saline conditions. Moreover, the improvement in antioxidant activity due to bacterial inoculation was non-significant with respect to absolute control under both saline and non-saline conditions.

### 2.6. Impact on Macro- and Micronutrient Content of Wheat

Salinity stress caused a significant reduction in the NPK content in the straw and grains of the wheat crop in all the treatments as compared to non-saline conditions (Table 4). The inoculation of *Bacillus* sp. strain AZ6 increased the NPK content of wheat straw and grains under both saline and non-saline conditions; however, an increase due to strain AZ6 was not statistically significant with respect to absolute controls under normal and saline conditions. In addition to this, the application of both BAZ- and ZnSO_4_-coated urea showed a significant increase in the NPK content of wheat straw and grains in normal as well as saline conditions. The BAZ-coated urea increased NPK content by up to 33, 36, and 37% in straw, while it was 39, 47, and 53% higher with ZnSO_4_-coated urea, over absolute control, under saline conditions. Similarly, the NPK content in grains increased by up to 36, 36, and 53% with BAZ-coated urea and 45, 55, and 50% with ZnSO_4_-coated urea when compared to absolute control under saline conditions.

**Figure 2:**
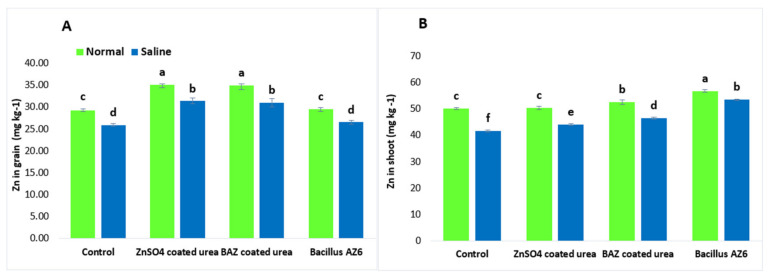
The Zn concentration in the shoots (**A**) and grains (**B**) of wheat grown with BAZ-coated urea under salinity stress. The presented data are the means of three replications (*n* = 3) ± standard error. The same alphabetical letters above error bars show that different treatments were not significantly different (*p* ≥ 0.05) from each other.

The Zn contents in the shoots and grains were similar in both the inoculated and un-inoculated absolute control plants under normal and saline conditions (Figure 2). Plants grown under saline conditions showed a significant reduction in Zn contents in the shoots and grains as compared to in non-saline conditions. The application of ZnSO_4_- and BAZ-coated urea ameliorated the salt stress and significantly increased the Zn contents in the shoots and grains; however, these treatments were not significantly different from each other but showed a significant increase as compared to absolute control. The application of BAZ-coated urea increased Zn contents in the shoots by up to 29.1% and in the grains by up to 16.5% under saline conditions, while the increases in the shoots and grains over their respective absolute controls were up to 33.0 and 16.2%, respectively (Figure 2).

### 2.7. Relationship and Variation among Morpho-Physio and Biochemical Attributes of Wheat

**Figure 3:**
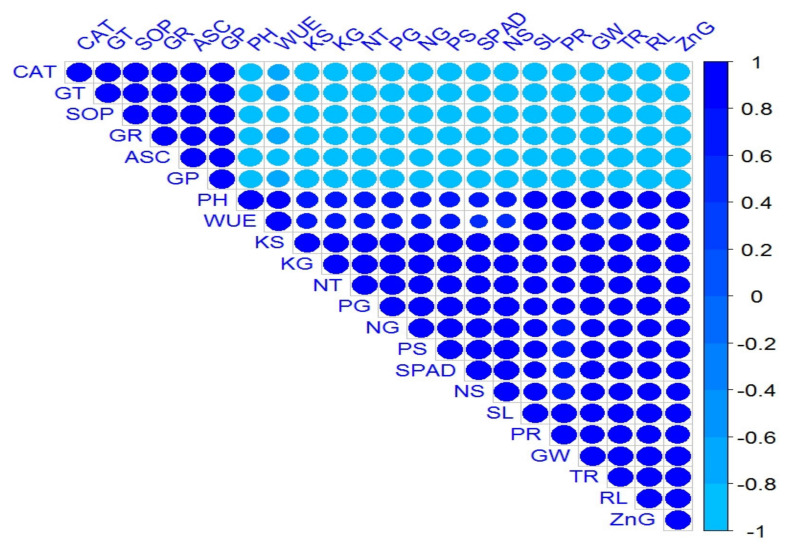
Corrplot representing correlation among measured attributes of wheat crop followed by treatments as Normal soil—(1) Control, (2) ZnSO_4_-coated urea, (3) BAZ-coated urea, and (4) *Bacillus* sp. strain AZ6—and Saline soil—(5) Control, (6) ZnSO_4_-coated urea, (7) BAZ-coated urea, and (8) *Bacillus* sp. strain AZ6. Positive correlations are displayed in royal blue, and negative correlations, in sky blue color. The color legend on the right-hand side of corrplot, shows correlation coefficients and the corresponding colors. The intensity of the color and circle size are proportional to the correlation coefficients. The abbreviations are as follows: PH = Plant height, RL = Root length, NT = No. of tillers, SL = Spike length, GW = 1000-grain weight, WUE = Water use efficiency (WUE), SPAD = Chlorophyll contents, PR = Photosynthetic rate, TR = Transpiration rate, CAT = Catalase, ASC = Ascorbate peroxidase, SOD = Superoxide dismutase, GR = Glutathione reductase, GT = Glutathione transferase, GP = Glutathione peroxidase, NG = Nitrogen in grain, NS = Nitrogen in straw, PG = Phosphorus in grain, PS = Phosphorus in straw, KG = Potassium in grain, KS = Potassium in straw, and ZnG = Zinc in grain.

Correlation analysis revealed a highly positive association among all the measured growth and yield parameters of the wheat plant, and its nutrient contents as well. Figure 3 shows a graphical display of the correlation matrix by corrplot. Similarly, highly positive correlations were also observed among all the measured antioxidant enzymes. Meanwhile, a significant negative association of antioxidant enzymes was observed with all the growth, productivity, and biochemical attributes of wheat.

**Figure 4:**
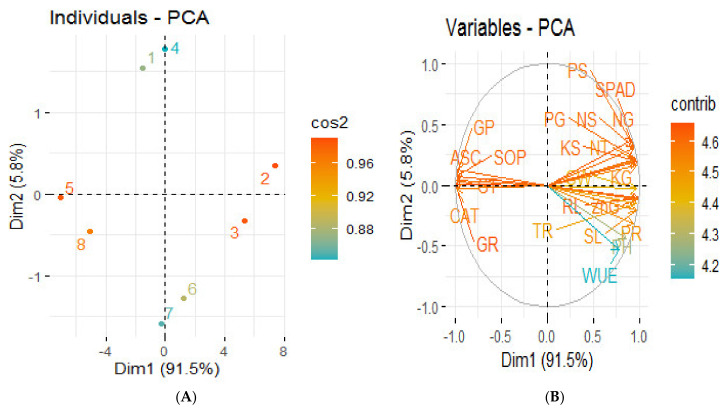
Principal component analysis (PCA) showing score plots (**A**) and loading plots (**B**) of different attributes of wheat plant under salt stress. Score plot (**A**) represents separation of treatments as Normal soil—(1) Control, (2) ZnSO_4_-coated urea, (3) BAZ-coated urea, and (4) *Bacillus* sp. strain AZ6—and Saline soil—(5) Control, (6) ZnSO_4_-coated urea, (7) BAZ-coated urea, and (8) *Bacillus* sp. strain AZ6. Loading plot (**B**) shows the loading of each studied variable (arrows), and the arrow lengths approximate their variance, whereas the angles between them represent their correlations. The abbreviations are as follows: PH = Plant height, RL = Root length, NT = No. of tillers, SL = Spike length, GW = 1000-grain weight, WUE = Water use efficiency, SPAD = Chlorophyll contents, PR = Photosynthetic rate, TR = Transpiration rate, CAT = Catalase, ASC = Ascorbate peroxidase, SOD = Superoxide dismutase, GR = Glutathione reductase, GT = Glutathione transferase, GP = Glutathione peroxidase, NG = Nitrogen in grain, NS = Nitrogen in straw, PG = Phosphorus in grain, PS = Phosphorus in straw, KG = Potassium in grain, KS = Potassium in straw, and ZnG = Zinc in grain. Dim: Dimension; Cos2: Square cosine; Contrib: Contribution.

The principal component analysis revealed the distribution of the different treatments performed on the wheat plant under saline conditions, as presented by the score plot (Figure 4A). Remarkable results were obtained from the score plot of PCA performed for two factors (cumulative variance, 97.3%); the first explains 91.5% of the variation, while 5.8% of the differences is explained by the second factor, thus showing great variation among the different treatments applied on the wheat plants. The maximum coordinate on the score plot of the PCA was obtained for ZnSO_4_-coated urea treatment, revealing it to be the most efficient treatment, followed by BAZ-coated urea in normal soil. The PCA loading plot (Figure 4B) shows a better visualization of the relationships and great variation present among all the studied growth and productivity parameters. It revealed that almost all the growth and yield attributes are positively correlated to each other with varying degrees of relationship, and comparatively less positively correlated to the analyzed macronutrients, while all these variables are negatively correlated to the antioxidant enzymes, which are positively correlated among themselves.

## 3. Discussion

Salt-affected soils having a high pH environment and calcium concentrations limit the Zn availability in the soil and cause Zn deficiency in crops. Such salt-affected soils also have dominant Na^+^ on exchange sites that cause Zn losses through a leaching process under irrigated conditions [15]. Generally, inorganic sources of Zn including ZnSO_4_ and ZnO are applied to fulfill the Zn demand in crops. The application of ZnSO_4_ in salt-affected soils is restricted due to the low Zn use efficiency and higher cost [27]. While ZnO is a cheaper source and contains 80% Zn, this concentration is insoluble in salt-affected and calcareous soils [28]. It is well-reported that Zn-solubilizing bacteria (ZSB) strains in soil have the power to solubilize insoluble Zn, and such available Zn could be termed as bioactivated Zn [25,26,29,30,31]. Hussain et al. [25,26] reported that bioaugmented ZnO promoted the Zn use efficiency as compared to conventional ZnO and ZnSO_4_ fertilizers. In the current study, we formulated bioactivated-Zn-coated urea by *Bacillus* sp. AZ6-augmented ZnO coating on urea and termed it as BAZ-coated urea. The effectiveness of BAZ-coated urea in promoting wheat physiology and productivity was evaluated under salt stress. The results revealed that the application of BAZ-coated urea alleviated the salt stress and significantly promoted the wheat physiology, growth, yield, and Zn uptake. Similar effects on plants could have been observed if the BAZ was not coated on urea but added separately to the soil. Urea coating with BAZ facilitates a one-time bacterium–Zn–urea application and eliminated the problem of the segregation of the smaller and larger nutrient particles in bulk fertilizer blends. Such improvement in the urea coating process may increase the efficiency of ZnO coating and improved the outcomes in terms of crop productivity and Zn availability.

In the current study, saline conditions inhibited the physiology, growth, and yield of wheat, which might be due to disruptions in membrane stability and photosynthetic activity, and an imbalance in nutrient uptake [32]. Bacterial inoculation and mineral application improved salt-stress tolerance in wheat [33]. Sohaib et al. [34] reported that the application of PGPR, e.g., *Pseudomonas fluorescens*, *Pseudomonas putida*, and *Serratia ficaria*, alleviated the salinity stress through their ACC-deaminase activity and promoted the physiology, growth, and yield attributes of wheat. The application of Zn minerals also alleviated the salinity stress through reducing Na^+^ and Cl^−^; increasing Zn and K^+^ concentrations; increasing the membrane stability index; increasing the SPAD value; increasing the activity of antioxidant enzymes including SOD, CAT, and GR; and enhancing phytohormones such as indole-3-acetic acid (IAA) and abscisic acid (ABA) [35]. Dimkpa et al. [22] demonstrated drought stress alleviation through the application of ZnO-nanoparticle-coated urea, which promoted wheat grain yield and Zn accumulation under drought stress. In the current study, we reported the alleviation of salt stress through the application of BAZ-coated urea and ZnSO_4_-coated urea. Under salt stress, both of these treatments were statistically similar in promoting wheat physiology, growth, and yield attributes but showed significantly high results as compared to the control treatment. BAZ-coated urea showed several benefits over ZnSO_4_-coated urea, as it is composed of ZnO augmented with *Bacillus* sp. AZ6, which is a promising Zn-solubilizing strain and enhances the solubility of ZnO [36]. Similar to our findings, Hussain et al. [25,26] also recommended the application of bioactivated ZnO to increase the productivity and biofortification of cereals.

In the present study, fertilization with BAZ-coated urea increased wheat physiology and growth under saline conditions, which might be due to an increase in Zn availability. Previously, several researchers reported an increase in the availability of Zn through the application of *Bacillus* spp. strains as potential bioinoculants [29,30,31,36,37]; however, the current study is novel regarding the formulation of a bioaugmented ZnO-coated urea product and its role in the alleviation of salt stress. Such bacterial strains have the power to solubilize the ZnO through producing volatile and non-volatile organic acids including acetic, caffeic, chlorogenic, cinnamic, citric, ferulic, formic, gallic, isobutyric, isovaleric, lactic, succinic, and syringic acids [30,36]. The increase in Zn availability under saline conditions improved the Zn status of the plant, leading to improved plant physiology. In the current study, the application of BAZ-coated urea might have promoted Zn availability, which increased the activity of carbonic anhydrase and other enzymes including ribulose 1,5-biphosphate carboxylase involved in photosynthetic activity, and increased chlorophyll contents [10,38]. The availability of Zn may also play a role in the metabolism of carbohydrates, sugars, and starches that could improve the stability of biomembranes under salt stress [10].

In the present study, the application of BAZ-coated urea under saline conditions promoted physiological attributes, e.g., the photosynthetic rate, transpiration ratio, stomatal conductance, SPAD value (Figure 1), and growth parameters, e.g., the germination rate, plant height, root length, and number of tillers (Table 1). This treatment also showed an increase in yield attributes including the number of grains, spike length, spike weight, and 1000-grain weight as compared to non-treated plants grown in saline conditions (Table 2). Such an increase in physiology, growth, and yield attributes under saline conditions might be due to an increase in N use efficiency that reduced the N losses through volatilization, denitrification, and leaching below the root zone and promoted the N uptake in the crops [39]. The increase in N availability alleviated the adverse effects of salt stress through promoting photosynthetic activity, stomatal conductance, total chlorophyll contents, the activity of carbonic anhydrase, and the accumulation of osmoprotectants and nutrients that promoted wheat growth and yield attributes [32]. Similarly, Hussain et al. [25,26] applied bioactivated organic Zn fertilizers and reported a significant increase in photosynthetic and transpiration rates, stomatal conductance, chlorophyll contents, carotenoids, carbonic anhydrase, plant height, dry shoot biomass, and Zn contents in shoots and grains, while showing a reduction in the phytate contents, of maize. The current study recommends that the application of BAZ-coated urea under salt stress can help wheat to alleviate the negative effects of salt stress and provide a better source of nutrients for plant growth.

Similarly, an increase in the magnitude of antioxidant enzyme activity (APX, GPX, GST, GR, CAT, and SOD) was observed, particularly, under saline conditions. These modifications of biochemical responses supported the findings of Bashir et al. [40], who reported an increase in the activities of antioxidants in *Zea mays* grown under tannery-polluted Cr soil. Sodium accumulation in plants alters several crucial cellular biomolecules’ activities, which consequently give rise to unnecessary reactive oxygen species (ROS) [41]. These ROS species become toxic, damage the membrane of the cell and its organelles, and cause cell death [42,43]. Consequently, plants, at the cost of their growth, resort to a series of mechanisms, i.e., a reduced accumulation of toxic Na, compartmentalization, and the synthesis of compatible solutes and antioxidant enzymes to resolve the ROS species [44,45]. The application of ZnSO_4_ and BAZ-coated urea remarkably lowered the activity of antioxidant enzymes, particularly under salinity stress. The accumulation of harmful Na might have replaced essential nutrients with functions, e.g., the replacement of K and Ca with Na altered stomatal opening and closing, and caused membrane disruption [46,47]. Zinc sulfate and BAZ-coated urea provided relief and lowered the antioxidant enzyme activity, which may be associated with enhancement of the nutrient uptake at the cost of Na accumulation in the plant [25]. An excessive Zn concentration in the soil probably lowered the uptake and accumulation of unnecessary Na [16,35].

The application of salinity stress lowered the contents of NPK in straw and grains. Excess Na^+^ in the saline environment perhaps lowered the uptake of essential NH_4_^+^ and K^+^ [48,49]. Similarly, excess Cl^−^ might have decreased the uptake of essential NO_3_^−^ and PO_4_^−^ [50,51]. An increase in the content of NPK in straw and grains was also observed with the application of both ZnSO_4_ and BAZ-coated urea. The addition of coated urea might have improved the soil organic matter and soil characteristics; the soil might have adsorbed the toxic Na by making it precipitates or complexes [44,52]. The reduced uptake and accumulation of Na in plant tissues might have lowered the cellular disruption and production of unnecessary ROS and have improved the plant photosynthesis due to the effective uptake of other essential nutrients [45,53].

The application of BAZ-coated urea promoted Zn and N availability for roots and reduced their fixation and leaching losses [27]. An increased uptake of Zn and N improves the root growth that enhances the nutrient uptake and accumulation in plant tissues. In the present study, the application of BAZ-coated urea in saline conditions promoted the uptake of Zn and resulted in improved Zn contents in wheat shoots and grains. The separate application of *Bacillus* sp. AZ6 coated on urea may acidify the environment through producing organic acids and chelating Zn as previously reported by Hussain et al. [25,26] and Mumtaz et al. [29]. This treatment might also cause improvement in the uptake and translocation of Zn from the roots, shoots, and grains [54]. A similar increase in grain Zn contents was also reported to be caused by *Bacillus* and *Pseudomonas* strains [55]. The increase in Zn contents in wheat grains might be due to the reduction in the anti-nutrient phytic acid caused by BAZ-coated urea. Cakmak et al. [56] reported that an increase in the Zn concentration due to a synthetic source or due to Zn-solubilizing strains causes a reduction in phytic acid. Previously, a reduction in the phytic acid concentration in grains was reported due to the application of Zn bioaugmented with *Pseudomonas* strain MN12 [57]. The application of BAZ urea at the farmer level will reduce the cost of production, as it will provide N as well as Zn to soils. This product will also improve fertilizer use efficiency. Further investigations regarding the field application of BAZ-coated urea under salt-affected conditions must be performed.

## 4. Materials and Methods

### 4.1. Preparation of Bacillus sp. Strain AZ6 Inoculum

Pre-isolated and pre-characterized *Bacillus* sp. strain AZ6 (accession number KT221633) [23] having the ability to convert ZnO into an exchangeable form (Zn^2+^) [29], was obtained from the Environmental Science Laboratory, Institute of Soil and Environmental Sciences (ISES), University of Agriculture Faisalabad (UAF), Pakistan. The strain AZ6 was grown in tris-minimal salt broth supplemented with 0.1% ZnO and incubated at 28 ± 1 °C for 48 h in a shaking incubator [24]. After incubation, the medium was centrifuged at 10,000 rpm and 22 °C for 20 min. The obtained bacterial pellet was suspended in sterile distilled water, and this was repeated until a cell load of 10^8^ cells mL^−1^ was achieved, determined by taking the optical density at 600 nm absorbance.

### 4.2. Production of Bacillus Augmented ZnO (BAZ)-Coated Urea

The bacterial pellet of strain AZ6 was suspended in sterile distilled water (0.65 mL) to obtain a bacterial cell load of 10^8^ cells mL^−1^ and mixed with black food coloring (0.07 mL). The BAZ-coated urea was prepared by augmenting dry fine bulk ZnO (0.72 g) powder with black-stained bacterial culture in a 50:50 (*w/v*) ratio (ZnO/AZ6) and incubating the mixture at 28 ± 1 °C for 72 h [19,20]. The BAZ was mixed with 2 mm-uniform-size urea (113 g) granules. The sterile water in the bacterial culture served as the binding agent, while the black food coloring provided contrast between the ZnO (80% Zn) powder and urea [22]. The resulting mixture was dried under room temperature for 72 h to achieve the maximum chelation of Zn with the AZ6 population. For comparison, commercially available zinc sulphate monohydrate (ZnSO_4_H_2_O; 35% Zn) was also coated on urea with black food coloring at the rate of 1.5% Zn and incubated for 72 h at 28 ± 1 °C. The mixing of urea with the Zn source was performed on a mechanical shaker at the low speed of 30 rpm. The control urea was coated with sterile distilled water and black food coloring and lacked the addition of ZnO powder and strain AZ6. After the preparation of the Zn-coated urea, the Zn concentrations, through an atomic absorption spectrophotometer, and N concentrations through Kjeldahl apparatus, were determined after an acid digestion method [58]. After the coating of the Zn sources on urea, the original urea coating (46% N) was 45.7% for ZnO and 45.8% for ZnSO_4_, while the original Zn content (1.5% Zn) was changed to 1.47% for ZnO and 1.48% for ZnSO_4_, indicating very negligible changes in the N and Zn contents during the coating process.

### 4.3. Soil Characterization

Before conducting the experiment, representative soil samples were taken from the field research area of ISES, UAF, at a 0–15 cm depth. These soil samples were air-dried, ground, sieved through a 2 mm-size mesh, and analyzed for soil physicochemical characteristics in terms of soil texture, saturation percentage, pH, electrical conductivity (EC), organic matter, the concentration of total nitrogen (N), available phosphorus (P), extractable K, and total Zn by following standard procedures. The particle size of the soil sediments was estimated to determine the texture class. A saturated soil paste was prepared and extracted through a vacuum pump for pH and electrical conductivity (EC) measurements using a digital pH meter (Kent-Eil 7015) and conductivity meter (Jenway, model 4070), respectively. The standard method of Moodie et al. [59] was followed to determine soil organic matter. Total nitrogen (N) was determined by using the Kjeldahl apparatus [60]. The available P, extractable K, and Zn concentrations were also estimated according to Jackson’s [60] method. To determine the total Zn concentration, the soil was extracted with 1 M ammonium bicarbonate (NH_4_HCO_3_) and 0.005 M diethylenetriamine penta acetate (DTPA), and the filtrate was then subjected to an atomic absorption spectrophotometer (AAS) (PerkinElmer, Analyst 100, Waltham, MA, USA).

### 4.4. Experimental Setup

The effects of the prepared BAZ-coated urea on wheat productivity were studied by performing a pot trial in the wire house of ISES, UAF, Pakistan. Thoroughly homogenized soil samples were used to fill the pots, 12 kg in each pot. The wheat crop cultivar Sahar-2006 was used as a test crop to evaluate the effect of urea coated with BAZ under salinity stress. Artificial salinity was developed by adding sodium chloride (NaCl) to the pots at two salinity levels: (i) non-saline and (ii) 100 mM saline conditions. The experiment was comprised of four treatments including T_1_ = absolute control, T_2_ = ZnSO_4_-coated urea, T_3_ = BAZ-coated urea, and T_4_ = *Bacillus* sp. strain AZ6 (without Zn). The treatments T_2_ and T_3_ were applied as soil applications of Zn, which were at the rate of 4.9 kg ha^−1^ and mixed thoroughly in the upper soil layer. Meanwhile, in the case of T_4_, wheat seeds were soaked in 48 h-old inoculum of strain AZ6 to measure the response of the ZSB strain only. For comparison, an absolute control (T_1_) was also maintained without any external source of Zn and strain AZ6. The seeds were sown in each pot, and after germination, six healthy plants were maintained. The pots were arranged in a completely randomized design (CRD) two-way factorial fashion in triplicate. The required doses of N (120 kg ha^−1^), P (90 kg ha^−1^), and K (60 kg ha^−1^) for wheat were applied by using urea, diammonium phosphate (DAP), and sulphate of potash (SOP) fertilizers, respectively. These fertilizers were applied at the time of sowing. All the treatments received the same dose of N as urea and DAP. Treatments 2 and 3 did not receive any additional N as urea and DAP. The N from the application of the BAZ-coated urea was also within the range of the recommended N dose. The plants were irrigated with tap water to maintain the optimum moisture for their growth. At the flowering stage, data regarding gas exchange parameters were observed. The crop was harvested after five months of sowing at the time of maturity, and data regarding the growth and yield attributes of the wheat were recorded.

### 4.5. Physiological Measurements

At the flowering stage, fully expanded second top leaves from each replication of treatments were selected and gas exchange parameters (the photosynthetic rate, transpiration rate, and stomatal conductance) were observed through the non-dispersive infrared gas analyzer (IRGA) of the Portable Photosynthesis (PP) System CIRAS-3 (PP System, Amesbury, MA, USA). The PLC3 narrow-leaf cuvette of the PP system CIRAS-3 was used to measure gas exchange attributes from both sides of the leaf. These measurements were made from 12.00 p.m. to 2.00 p.m. with the specifications reported by Mumtaz et al. [32,61]. For the determination of leaf chlorophyll contents, a chlorophyll meter (Konica Minolta sensing, Inc., Osaka, Japan) was used and measurements were taken after the selection of three leaves from each plant.

### 4.6. Determination of Growth Attributes

The germination percentage was estimated after sowing the seeds in pots. At the harvesting stage, growth attributes including the plant height, root length, and number of tillers were recorded. Plant height was measured with the help of a measuring rod from top to bottom for all the tillers in a plant and averaged. For the measurement of root length, plants were first uprooted and cleaned by washing with tap water and then the root lengths of all the roots in a plant were recorded with the help of a meter rod and averaged. Three plants in each pot were manually counted to observe the number of tillers per plant.

### 4.7. Antioxidant Enzyme Activity

The antioxidant enzymes were extracted by homogenizing frozen fresh leaf material in an ice-cold solution containing potassium phosphate buffer (0.2 M, pH 7) having 0.1 mM Ethylenediaminetetraacetic acid (EDTA). Ascorbate peroxidase (APX) activity was determined by tracking ascorbate reduction through H_2_O_2_ with a decrease in spectrophotometer absorbance at 290 nm [62]. Glutathione peroxidase (GPX) activity was determined according to the method of Aebi and Bergmeyer [63]. Glutathione-S-transferase (GST) activity was determined by following Habig et al. [64]. Glutathione reductase (GR) activity was measured by an increase in spectrophotometer absorbance at 412 nm observed due to the reduction of 5,5′-dithiobis 2-nitrobenzoic acid (DTNB) into 2-nitro-5-thiobenzoic acid (TNB) following the method of Smith et al. [65]. For catalase (CAT), 2 mL of 200-fold-diluted enzyme extract in potassium phosphate buffer (50 mM, pH 7.0) and 1 mL of H_2_O_2_ (10 mM) were used, following the method of Cakmak and Marschner [66]. The superoxide dismutase (SOD) activity was monitored with one milliliter of reaction mixture that contained 50 mM sodium phosphate buffer (pH 7.8), 100 mM EDTA, 20 mL of enzyme extract, and 10 mM pyrogallol according to the method illustrated by Roth and Gilbert [67].

### 4.8. Measurement of Wheat Yield

The yield attributes in terms of the spike weight, the number of grains per spike, and the 1000-grain weight were determined at the time of harvesting. Spikes were weighed to observe spike weight, and their grains were manually separated and counted to report the number of grains per spike. The weight of 100 grains from each replication was recorded with a digital weighing balance and multiplied by 10 to obtain the 1000-grain weight.

### 4.9. Analysis of Macro- and Micronutrients

Plant shoot and grain samples were finely grounded with a Wiley mill fitted with a stainless steel chamber and blades. Ground plant samples (0.2 g) were digested according to the method described by Wolf [58]. After digestion, the final volume was made up to 50 mL with deionized water. The nitrogen content was determined with the Kjeldahl method [60], and P was measured on a UV–visible spectrophotometer after developing yellow color by the vanadate–molybdate method [68] at 410 nm using a standard curve. A flame photometer was used for K determination in plant samples using a standard curve.

The concentration of Zn in wheat shoot and grain samples was measured by the wet digestion method. Air-dried, l g ground samples were taken in a digestion flask along with 10 mL of concentrated HNO_3_ and incubated overnight. After incubation, the samples were heated on a hot plate until the production of red NO_2_ fumes had ceased. Furthermore, 2–4 mL of 70% HCIO_4_ was added and again heated up to the colorless endpoint. The digested samples were filtered and diluted up to 50 mL in a flask. A clear filtrate was used to determine the Zn concentration with an AAS after plotting a standard curve with the help of calibrated working standards of Zn (0.5, 1.0, 1.5, 2.0, 2.5, and 3.0 ppm).

### 4.10. Statistical Analysis

A statistical technique was established to evaluate the effect of BAZ-coated urea on wheat performance. The treatments were randomly divided into smaller groups and end tasks in terms of wheat physiology, growth, and yield attributes and were analyzed through two-way analysis of variance (ANOVA) by employing a linear CRD factorial design in the computer-based software Statistix v. 8.1 (Analytical Software, Tallahassee, FL, USA). The treatment means were compared by applying the least significant difference (LSD) test at a 5% probability level [69]. The relationships among all the studied parameters of the wheat plant were determined by Spearman’s correlation analysis, performed in the R program (version 2.3.1). Principal component analysis (PCA) was also performed to compare the effect of BAZ-coated urea with the effects of other applied treatments on the physiology and productivity of wheat under salt stress.

## 5. Conclusions

In the current study, the effects of BAZ-coated urea on wheat physiology, growth, yield, antioxidant activity, NPK content, and Zn uptake were studied, under saline conditions. The application of BAZ-coated urea under salinity stress significantly improved wheat productivity by improving physiological attributes and Zn uptake. Here, BAZ-coated urea counteracted salt stress by improving the wheat germination rate, plant height, root length, photosynthetic rate, transpiration rate, stomatal conductance, chlorophyll contents, spike length, grain number, grain weight, antioxidant activity (APX, GPX, GST, GR, CAT, and SOD), NPK contents in the straw and grains, and Zn concentration in the shoots and grains. Moreover, such potential treatments may improve soil fertility through enhancing the availability and uptake of Zn in wheat by chelating the insoluble Zn fractions. In this way, the farming community can obtain double benefits for salt-affected soils by applying urea coated with BAZ, which not only ameliorates nutrient deficiencies in the plant and soil but also enriches wheat grains with Zn to meet humans’ nutritional requirements.

## Figures and Tables

**Table 1:** The impact of BAZ (*Bacillus* augmented ZnO)-coated urea on growth parameters of wheat under salinity stress.

Treatments	Salinity Conditions	Plant Height (cm)	Root Length (cm)	Germination Rate (%)	No. of Tillers Plant^−1^
Control	Normal	65.4 ± 1.2 c	22.0 ± 0.44 d	74.3 ± 1.91 c	3.3 ± 0.38 bcd
	Saline	59.3 ± 0.9 d	19.3 ± 0.27 e	65.7 ± 2.10 d	2.3 ± 0.33 d
ZnSO_4_ coated urea	Normal	81.1 ± 1.1 a	27.4 ± 0.61 a	92.3 ± 2.45 a	4.6 ± 0.37 a
	Saline	79.7 ± 1.2 a	24.1 ± 0.24 b	82.7 ± 2.24 b	3.7 ± 0.37 abc
BAZ-coated urea	Normal	80.0 ± 0.8 a	26.7 ± 0.38 a	90.7 ± 2.35 a	4.0 ± 0.58 ab
	Saline	71.3 ± 0.9 b	23.5 ± 0.43 bc	80.0 ± 3.07 bc	3.0 ± 0.12 bcd
*Bacillus* sp. strain AZ6	Normal	78.6 ± 0.3 a	22.6 ± 0.47 cd	80.0 ± 1.86 bc	3.7 ± 0.21 abc
	Saline	68.6 ± 0.8 b	19.8 ± 0.18 e	72.3 ± 1.41 cd	2.7 ± 0.32 cd

The presented data are the means of three replications (*n* = 3) ± standard error. The mean values sharing the same letters were considered not significantly different from each other at *p* ≥ 0.05.

**Table 2:** The impact of BAZ (*Bacillus* augmented ZnO)-coated urea on yield parameters of wheat under salinity stress.

Treatments	Salinity Conditions	1000-Grain Weight (g)	Spike Length (cm)	Spike Weight (g)	No. of Grains Spike^−1^
Control	Normal	38.63 ± 0.42 b	8.17 ± 0.44 de	12.7 ± 1.14 c	35.7 ± 1.14 d
	Saline	33.83 ± 0.46 c	7.33 ± 0.17 e	10.5 ± 0.93 d	30.0 ± 1.64 e
ZnSO_4_	Normal	44.93 ± 1.44 a	11.26 ± 0.44 a	16.7 ± 1.05 a	48.7 ± 2.32 a
coated urea	Saline	40.06 ± 0.18 b	9.50 ± 0.29 bc	14.0 ± 1.21 b	42.0 ± 1.87 b
BAZ-coated urea	Normal	44.01 ± 1.34 a	10.67 ± 0.17 ab	15.7 ± 0.88 a	47.3 ± 2.75 a
	Saline	39.73 ± 1.08 b	9.01 ± 0.58 cd	13.8 ± 0.75 b	40.7 ± 2.36 bc
*Bacillus* sp. strain AZ6	Normal	38.13 ± 0.71 b	8.16 ± 0.60 de	12.9 ± 1.01 c	37.3 ± 1.95 cd
	Saline	32.83 ± 0.55 c	7.50 ± 0.31 e	10.9 ± 0.64 d	31.3 ± 2.16 e

The presented data are the means of three replications (*n* = 3) ± standard error. The mean values sharing the same letters were considered not significantly different from each other at *p* ≥ 0.05.

**Table 3:** The impact of BAZ (*Bacillus* augmented ZnO)-coated urea on antioxidant enzyme activity of wheat under salinity stress.

Treatments	Salinity Conditions	Ascorbate Peroxidase (nmol mint^−1^ g^−1^)	Glutathione Peroxidase (nmol mint^−1^ g^−1^)	Glutathione Transferase (µmol mint^−1^ mg^−1^)	Glutathione Reductase (nmol mint^−1^ mg^−1^)	Catalase (nmol mint^−1^ mg^−1^)	Superoxide Dismutase (nmol mint^−1^ mg^−1^)
Control	Normal	31.2 ± 1.41 b	50.0 ± 1.75 c	197.0 ± 6.85 c	20.7 ± 0.95 b	9.88 ± 0.48 c	134.0 ± 5.55 bc
	Saline	45.1 ± 1.78 a	74.6 ± 1.52 a	297.0 ± 9.34 a	30.3 ± 1.23 a	15.4 ± 0.52 a	189.0 ± 6.91 a
ZnSO_4_ coated urea	Normal	12.8 ± 1.06 d	19.9 ± 1.38 e	63.0 ± 4.44 d	7.5 ± 0.73 c	3.68 ± 0.35 d	55.0 ± 4.43 f
	Saline	22.2 ± 1.24 c	35.5 ± 1.72 d	136.0 ± 4.14 d	16.6 ± 0.77 b	8.0 ± 0.40 c	98.0 ± 4.65 de
BAZ-coated urea	Normal	14.7 ± 0.71 d	26.4 ± 1.34 e	105.0 ± 5.02 d	9.5 ± 0.87 c	4.67 ± 0.36 d	75 ± 5.10 ef
	Saline	25.3 ± 1.43 bc	43.1 ± 1.58 cd	175.0 ± 7.23 c	19.0 ± 0.99 b	8.6 ± 0.40 c	115.0 ± 4.36 cd
*Bacillus* sp. strain AZ6	Normal	29.4 ± 1.27 b	46.1 ± 1.97 c	177.0 ± 8.66 c	18.7 ± 1.02 b	7.92 ± 0.43 c	119.0 ± 5.99 cd
	Saline	40.0 ± 1.59 a	64.3 ± 1.89 b	259.0 ± 8.86 b	26.5 ± 1.07 a	12.6 ± 0.47 b	161.0 ± 6.33 b

The presented data are the means of three replications (*n* = 3) ± standard error. The mean values sharing the same letters were considered not significantly different from each other at *p* ≥ 0.05.

**Table 4:** The impact of BAZ (*Bacillus* augmented ZnO)-coated urea on macronutrient contents of wheat under salinity stress.

Treatments	Salinity Conditions	Nitrogen in Straw (g kg^−1^)	Nitrogen in Grains (g kg^−1^)	Phosphorous in Straw (g kg^−1^)	Phosphorous in Grains (g kg^−1^)	Potassium in Straw (g kg^−1^)	Potassium in Grains (g kg^−1^)
Control	Normal	15.2 ± 0.65 c	11.0 ± 0.42 cd	1.62 ± 0.06 cd	0.83 ± 0.03 c	12.3 ± 0.51 cd	8.5 ± 0.31 c
	Saline	10.5± 0.38 e	7.2 ± 0.36 f	1.07 ± 0.05 f	0.54 ± 0.02 e	8.7 ± 0.45 e	6.1 ± 0.30 e
ZnSO_4_ coated urea	Normal	20.5 ± 0.58 a	15.8 ± 0.54 a	2.29 ± 0.07 a	1.19 ± 0.05 a	17.7 ± 0.69 a	12.7 ± 0.47 a
	Saline	14.6 ± 0.44 cd	10.5 ± 0.41 cde	1.57 ± 0.07 de	0.84 ± 0.04 cd	13.3 ± 0.68 bc	9.1 ± 0.37 bc
BAZ-coated urea	Normal	19.4 ± 0.51 ab	13.7 ± 0.37 ab	2.07 ± 0.05 ab	1.05 ± 0.04 ab	15.8 ± 0.54 ab	10.6 ± 0.43 b
	Saline	14.0 ± 0.38 cd	9.8 ± 0.33 de	1.45 ± 0.05 de	0.74 ± 0.04 cd	11.9 ± 0.59 cd	8.0 ± 0.39 cd
*Bacillus* sp. strain AZ6	Normal	17.5 ± 0.52 b	12.5 ± 0.36 bc	1.87 ± 0.07 bc	0.89 ± 0.03 bc	14.3 ± 0.47 bc	9.1 ± 0.33 bc
	Saline	11.7 ± 0.48 de	8.5 ± 0.31 ef	1.29 ± 0.04 f	0.66 ± 0.03 de	10.0 ± 0.51 de	6.7 ± 0.28 de

The presented data are the means of three replications (*n* = 3) ± standard error. The mean values sharing the same letters were considered not significantly different from each other at *p* ≥ 0.05.
